# Characteristics and Outcome of Patients with or without Previous Implantable Cardioverter Defibrillator Interventions Undergoing Ablation for Ventricular Tachycardia

**DOI:** 10.3390/jcm13164958

**Published:** 2024-08-22

**Authors:** Gianluigi Bencardino, Maria Lucia Narducci, Roberto Scacciavillani, Francesca Augusta Gabrielli, Gemma Pelargonio, Massimo Massetti, Filippo Crea, Gaetano Antonio Lanza

**Affiliations:** 1 Department of Cardiovascular and Thoracic Sciences, Fondazione Policlinico Universitario A. Gemelli IRCCS, 00168 Rome, Italy; marialucia.narducci@policlinicogemelli.it (M.L.N.); roberto.scacciavillani@gmail.com (R.S.); francescaaugusta.gabrielli@policlinicogemelli.it (F.A.G.); gemma.pelargonio@policlinicogemelli.it (G.P.); massimo.massetti@policlinicogemelli.it (M.M.); filippo.crea@unicatt.it (F.C.); gaetanoantonio.lanza@policlinicogemelli.it (G.A.L.); 2 Cardiology Institute, Catholic University of Sacred Heart, 00136 Rome, Italy

**Keywords:** ventricular arrhythmias, catheter ablation, electrical storm

## Abstract

**Background:** Catheter ablation (CA) is a well-established treatment in patients with ventricular tachycardia and appropriate implantable cardioverter defibrillator (ICD) therapies. **Methods:** We enrolled 57 consecutive carriers of ICD undergoing CA for electrical storm (ES). Our aim was to investigate differences in clinical, device-related, and electroanatomic features among patients who had history of appropriate ICD interventions before the ES compared to those who had not. The primary endpoint was a composite of death from any cause and recurrences of sustained VT, ventricular fibrillation, appropriate ICD therapy, or ES. **Results:** During a median follow up of 39 months, 28 patients (49%) met the primary endpoint. Those with previous ICD interventions had a higher prevalence of late potentials and a greater unipolar low-voltage area at electroanatomic mapping. Patients who met the primary endpoint had a higher prevalence of ATP/shock episodes preceding the ES event. At Cox regression analysis, non-ischemic dilated cardiomyopathy (NIDCM), QRS duration, and previous ATP and/or shock before the ES were associated with arrhythmic recurrences and/or death. At multivariate analysis, NIDCM and previous shock were associated with arrhythmic recurrences and/or death. **Conclusions**: A history of recurrent ICD therapies predicts worse outcomes when CA is needed because of ES. Although more studies are needed to definitively address this question, our data speak in support of an early referral for CA of ES.

## 1. Introduction

Electrical storm (ES) is characterized by three or more distinct episodes of ventricular arrhythmia within 24 h [1]. ES is a medical emergency that portends a significant increase in mortality risk and often presages progressive heart failure. The mechanism of ES remains elusive, but is likely influenced by a complex interplay of inciting triggers (e.g., ischemia, electrolyte disturbances), with autonomic perturbations acting on a vulnerable structural and electrophysiologic substrate. Catheter ablation (CA) is a well-recognized therapy in such patients, and it is proven to be associated with an improvement in prognosis [2,3].

On the other hand, patients with an implanted cardioverter defibrillator (ICD) who present sporadic interventions of the device are usually managed conservatively, although growing evidence suggests that the composite endpoint of death/arrhythmia recurrence can be lower among those undergoing CA and receiving an escalation in antiarrhythmic drug therapy [4].

The primary aim of this study was to investigate whether, among ICD patients presenting with ES, differences in clinical outcome exist between those with vs. those without appropriate ICD interventions before the ES event.

## 2. Methods

### 2.1. Study Design and Patient Selection

This was a single-center prospective observational study that involved the enrolment of 57 consecutive patients with a previous ICD implantation for the primary or secondary prevention of sudden cardiac death, referred for CA, who fulfilled the following inclusion criteria: (1) at least 1 episode of ES, defined as 3 or more episodes of sustained ventricular tachycardia (VT) or ventricular fibrillation (VF) with appropriate ICD shocks in 24 h; (2) acute procedural success of CA, defined as the abolition of all abnormal electrograms at electroanatomic mapping and no inducibility of VT/VF by programmed ventricular stimulation [5]. ICDs after CA were programmed with a 2-zone configuration and long delay, according to recent consensus indications [6]: (1) a VT zone ranging from 170 to 200 bpm that allowed up to 3 interventions with anti-tachycardia pacing (ATP), followed by ICD shock if ATP was unsuccessful; (2) a VF zone that started from 200 bpm and allowed shock interventions with 1 ATP during charging time (up to 6 times for each episode).

### 2.2. Catheter Ablation Procedures

All patients underwent endocardial and/or epicardial ventricular high-density electroanatomic mapping and ablation for ES. Electroanatomic substrate mapping was performed in all patients with bipolar and unipolar voltages defined as the peak-to-peak electrogram voltage. Dense scar was defined as bipolar voltage <0.5 millivolts (mV). Low-voltage area was defined as a bipolar voltage <1.5 mV and a unipolar voltage <8.3 mV, as previously described [7]. Local abnormal ventricular activity (LAVA) areas were defined as the presence of fractionated electrograms within the QRS complex, whereas late potentials (LPs) were defined as the presence of fractionated electrograms after the end of the QRS complex [8]. Programmed stimulation was performed at the initial stage of the ablation procedure at 2 drive trains (600 ms and 400 ms) with up to triple extrastimuli down to ventricular effective refractory period or 200 ms. Activation and entrainment mapping were performed if induced VTs were hemodynamically stable. A substrate-based ablation strategy targeting abnormal regions of slow conduction was performed in the event of non-inducibility or in hemodynamically unstable VTs. Irrigated ablation technology was used for ablation, with the choice of the catheter left to the discretion of the physician performing the procedure. The immediate endpoint of CA was the abolition of abnormal electrograms (LAVA and LPs) and the non-inducibility of VT/VF, with a complete programmed electrical stimulation protocol, performed with at least the same number and extent of extrastimuli and coupling intervals as those applied for the induction sequence before CA [9]. Remapping was performed at the end of the procedure to verify the abolition of abnormal electric potentials. 

### 2.3. Clinical and Device Follow Up

Clinical data, including age, gender, cardiovascular risk factors, past medical history, medication profiles, electrocardiograms, transthoracic echocardiograms, and electroanatomic mapping data, were obtained from all patients. Clinical and device follow-up was carried out at 3–6 month intervals (or before if clinically indicated) and up to 48 months after CA. The appropriateness of all ICD interventions was adjudicated by an expert electrophysiologist. All patients signed a dedicated informed consent form to agree to the study procedure, which included authorization for database insertion and clinical follow-up assessment. The study was approved by the Institutional Committee on Human Research of our institution.

### 2.4. Patient Groups and Study Endpoint

The patients were divided into 2 groups: (1) patients with a history of one or more appropriate ICD interventions due to sustained VT/VF before the index episode of ES and CA (Group 1); and (2) patients without any history of previous ICD intervention before ES/CA (Group 2). We cannot exclude the possibility that Group 2 patients could have undergone previous pharmacological or electrical cardioversion of VT/VF before device implantation. However, no evidence of further ventricular events was found after device implantation.

The primary endpoint of the study was a composite of death from any cause, recurrence of ES, or occurrence of sustained VT or VF with appropriate ICD interventions.

### 2.5. Statistical Analysis

Continuous variables are expressed as mean ± standard deviation or median with interquartile range (for non-normally distributed variables). Comparisons between the 2 groups were carried out by an unpaired Student’s *t*-test or Mann–Whitney U-test, as indicated, whereas categorical variables were compared by a chi-square test. Univariate and multivariable Cox regression analyses were performed to identify independent predictors of the primary endpoint. Variables with a *p*-value ≤ 0.05 on univariate analyses were included as independent covariates in the multivariable model. Survival curves were constructed using the Kaplan–Meier method and compared by a log-rank test. A *p*-value < 0.05 was always required for statistical significance. All analyses were performed using SPSS Statistics 23.0 software (SPS Inc., Florence, Italy).

## 3. Results

### 3.1. Characteristics of Patients

From January 2017 to January 2022, 57 patients were found who fulfilled the inclusion criteria for the study. The patients had undergone ICD implantation 32 ± 21 months before enrollment, 37 (65%) of which were for primary prevention.

Forty patients (70%) had evidence of appropriate ICD interventions before the ES/CA (Group 1), whereas 17 did not (Group 2).

In the Group 1 patients, the first occurrence of ICD intervention occurred 13 ± 5 months before the ES/CA. Furthermore, the number of appropriate ICD interventions for VT/VF before ES/CA was 4.6 ± 3.8.

The main clinical characteristics of the two groups of patients are summarized in Table 1a,b. As shown, the patients in Group 1 had a lower EF, similar rates of beta-blocker use before and after CA, a higher percentage of amiodarone use before ES but not after CA, and a longer duration of QRS and QT interval. The procedural times did not differ significantly between groups. Eight (14%) procedures were completed under general anesthesia, and the others under local anesthesia and sedation. Hemodynamic support with venoarterial extracorporeal membrane oxygenation (VA-ECMO) was needed in five subjects (9%). Endocardial mapping was performed in every patient, while an epicardial approach was needed in 14 (25%) and activation mapping was possible in 51 (89%). Group 1 patients also had a higher prevalence of LPs and a larger area of low unipolar voltages. Peri- and post-procedural major complications, including cardiac perforation with or without tamponade, systemic thromboembolism, or vascular access injury requiring intervention, are reported in Table 1b.

### 3.2. Predictors of Arrhythmic Recurrence and/or Death after CA for ES

**Figure 1:**
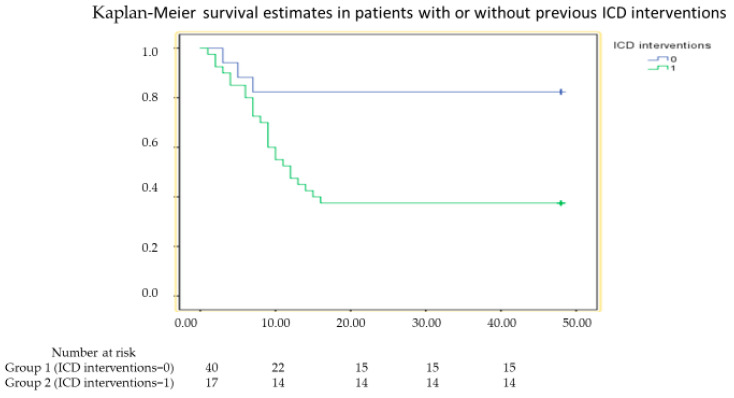
Kaplan–Meier survival curve in patients with or without appropriate implantable cardioverter defibrillator (ICD) shock who met the primary endpoint of death or ventricular arrhythmia recurrence (log rank < 0.001).

During a median follow up of 39 months (range 8–48 months), 28 patients (49%) met the primary endpoint of the study. Clinical events occurred in 25 (63%) and 3 (18%) patients in Group 1 and Group 2, respectively (hazard ratio [HR] 14.12, confidence interval [CI] 4.32–46.15, *p* < 0.001). The Kaplan–Meier curves of the two groups of patients are shown in Figure 1. Of these 28 subjects, 5 died (4 in Group 1 and 1 in Group 2, *p* = 0.62) and all had VAs (25 in Group 1 and 3 in group 2, *p* = 0.002), but only 19 received appropriate shock on VAs (16 in Group 1 and 3 in Group 2, *p* = 0.1).

The results of univariate and multivariate Cox regression analyses are summarized in Table 2. Non-ischemic dilated cardiomyopathy, QRS duration, and previous ICD interventions were the only variables associated with the endpoint, but only NIDCM (HR 4.59, CI 1.83–11.53, *p* = 0.001) and previous ICD interventions (HR was 14.12, CI 4.32–46.15, *p* < 0.001) maintained an independent association with the end point at multivariate regression analysis.

## 4. Discussion

Two main findings emerge from our data: (1) in patients undergoing CA for ES, a history of previous ICD interventions is associated with a higher occurrence of the composite endpoint of death/arrhythmia recurrence during follow-up; and (2) this difference seems to be related to a wider and more complex electroanatomic substrate, since patients with previous appropriate ICD interventions had larger unipolar scar areas and longer QRS and QT interval duration CA in patients presenting with ES. On the other hand, the indication to CA in patients presenting with a first episode of VT or sporadic episodes of VT/VF appropriately terminated by an ICD with ATPs and/or shocks remains a controversial topic. The most recent guidelines recommend that, in patients with ischemic heart disease who experience recurrent, symptomatic sustained monomorphic VT or ICD shocks for VT despite chronic amiodarone therapy, CA should be preferred to escalating antiarrhythmic drug therapy (class I/B recommendation) [1]. In contrast, in patients with non-ischemic dilated cardiomyopathy, CA should only be considered in specialized centers and only when antiarrhythmic therapy is ineffective, contraindicated, or not tolerated (class IIa/C recommendation) [1]. However, evidence from randomized clinical trials (RCTs) about this point is scarce and heterogeneous. In addition, after deciding to perform CA, the timing remains a matter of debate. We performed the procedures in the first 24 to 48 h after the admissions, but some authors suggest waiting >48 h in high-risk patients [10].

The SMASH-VT trial (Substrate Mapping and Ablation in Sinus Rhythm to Halt Ventricular Tachycardia) showed that CA reduced ICD therapies in patients with ischemic heart disease and sustained VT, but had no effect on mortality [11]; similar results were obtained by the VTACH (Substrate Modification in Stable Ventricular Tachycardia in Addition to Implantable Cardioverter Defibrillator Therapy) trial [12]. The VANISH trial (Ventricular Tachycardia Ablation versus Escalation of Antiarrhythmic Drugs), however, found that CA of VT in patients with ischemic cardiomyopathy was superior to escalating antiarrhythmic drugs in reducing the composite primary end point of death, AS, or appropriate ICD shock [4]. In the more recent SMS (Substrate Modification Study)-VT trial, instead, no differences in terms of VT recurrences were found in patients with and without CA [13]. A similar result was obtained in the Berlin VT study, which was, in fact, interrupted due to an increase in the primary endpoint (all-cause death, hospitalization for worsening HF or arrhythmia) in the CA arm [14]. On the other hand, more recently, the SURVIVE-VT trial reported that CA reduced the occurrence of the composite endpoint of cardiovascular death, appropriate ICD shock, hospitalization due to heart failure, or severe treatment-related complications compared to the AAD in patients with ischemic cardiomyopathy and symptomatic VT [15]. Similarly, the PARTITA trial CA was associated with a reduced occurrence of death or hospitalization for worsening heart failure and fewer ICD shocks, compared to standard treatment, in ischemic patients with previous ICD shocks for VT [9]. Finally, in the PAUSE-SCD trial, CA significantly reduced the rates of the composite primary outcome of VT recurrence, cardiovascular hospitalization, or death in patients with cardiomyopathy of varied causes and VT, although this result was mainly driven by a reduction in ICD therapies [16]. This complicated scenario raises important questions regarding the optimal timing of CA for VT. The data from our study speak in support of an early referral for CA of ES and raise the question of whether an early referral for VT ablation in patients with isolated or few ICD interventions may yield better outcomes. It is indeed possible that CA might, in this case, eliminate the electro-anatomic substrate, or part of the substrate, that would be responsible for the occurrence of ES during follow-up.

On the other hand, performing CA late, after a long history of conservative treatment, renders CA inevitably less effective in preventing arrhythmia recurrences, as patients tend to develop a more compromised electroanatomic substrate over time.

In patients with atrial fibrillation (AF), the importance of performing CA before atrial enlargement develops is well known, as it is known that recurrences of AF and changes from paroxysmal to persistent/permanent AF result in atrial enlargement, thus making the electro-anatomic substrate of the arrhythmias less responsive to CA [17,18]. Experience derived from AF ablation might be also appropriate for VT patients. It might indeed be speculated that recurrences of VT might contribute to the occurrence of adverse electro-anatomical remodeling, which eventually renders CA for arrhythmia prevention less effective. Randomized controlled trials, however, are needed to assess this hypothesis.

Our study has some inherent limitations. First, it includes a small sample of patients and is monocentric. Second, it is only an observational study and, therefore, the observation needs to be confirmed in randomized controlled studies. Finally, our population was heterogeneous because we enrolled patients with ES regardless of the underlying heart disease (ICM or NIDCM) and type of antiarrhythmic therapy.

## 5. Conclusions

A history of previous ICD therapies in patients undergoing CA for ES is associated with a worse clinical outcome. Large RCTs are needed to test the effects of an early referral of CA for ES.

## Figures and Tables

**Table 1:** (**a**) Comparison of clinical characteristics in patients with and without appropriate ICD interventions before the ES. (**b**) Comparison of electrocardiographic and procedural characteristics in patients with and without appropriate ICD interventions before the ES.

**(a)**			
	**Group 1** **(*n* = 40)**	**Group 2** **(*n* = 17)**	***p***
Age	73 ± 4	73 ± 5	0.74
Males	37 (93%)	17 (100%)	0.25
Diabetes mellitus	10 (25%)	4 (24%)	0.91
NYHA class	1.8 ± 0.8	1.8 ± 0.6	0,44
NSVT pre-ES	20 (50%)	7 (42%)	0.54
LVEF (%)	34 ± 11	43 ± 9	0.004
Moderate–severe VHD	6 (15%)	1 (6%)	0.34
NIDCM	15 (38%)	3 (18%)	0.11
Primary prevention	29 (73%)	8 (47%)	0.07
Amiodarone pre	29 (73%)	3 (18%)	<0.0001
Beta-blockers pre	39 (98%)	15 (88%)	0.15
Amiodarone post	14 (35%)	3 (18%)	0.19
Beta-blockers post	38 (95%)	15 (88%)	0.36
AF	13 (33%)	3 (18%)	0.25
PAINESD score	16.4 ± 4	17.7 ± 3.6	0.23
**(b)**			
	**Group 1** **(*n* = 40)**	**Group 2** **(*n* = 17)**	***p***
HR (bpm)	68 ± 14	67 ± 13	0.88
QRSd (msec)	159 ± 32	109 ± 17	<0.0001
QT (msec)	455 ± 47	423 ± 36	0.015
QTc (msec)	472 ± 49	438 ± 32	0.003
Mapping and ablation			
Procedural time (min)	201 ± 18	199 ± 20	0.69
General anesthesia	6 (15%)	2 (12%)	0.72
Hemodynamic support	4 (10%)	1 (6%)	0.62
Epicardial approach	12 (30%)	2 (12%)	0.14
Activation mapping	37 (93%)	14 (82%)	0.25
Unipolar low voltage	59 ± 42	30 ± 22	0.001
Bipolar scar	29 ± 24	17 ± 20	0.08
LPs/LAVA	28 (70%)	7 (41%)	0.041
Major complications	3 (8%)	1 (6%)	0.83

Abbreviations: ICD: implantable cardioverter defibrillator; ES: electrical storm; NYHA: New York Heart Association; NSVT: non-sustained ventricular tachycardia; LVEF: left ventricle ejection fraction; VHD: valvular heart disease; NIDCM: non-ischemic dilated cardiomyopathy; AF: atrial fibrillation. Abbreviations: HR: heart rate; LPs: late potentials; LAVA: local abnormal ventricular activity.

**Table 2:** Univariate and multivariable analyses of predictors of recurrence of VA and/or death.

		Univariate Analysis			Multivariable Analysis	
	HR	95% CI	*p*	HR	95% CI	*p*
Age	0.95	0.86–1.04	0.24			
Sex (Male)	0.58	0.14–2.42	0.45			
Diabetes mellitus	0.97	0.41–2.28	0.94			
NYHA class	0.99	0.57–1.75	0.99			
NSVT	0.73	0.35–1.55	0.42			
LVEF (%)	0.98	0.95–1.02	0.37			
Amiodarone	1.65	0.76–3.58	0.20			
NIDCM	3.21	1.42–7.22	0.005	4.59	1.83–11.5	0.001
AF	1.63	0.75–3.53	0.22			
QRSd (msec)	1.01	1.00–1.02	0.04	1.00	0.99–1.01	0.88
QT (msec)	1.01	0.99–1.02	0.17			
QTc (msec)	1.00	0.99–1.01	0.46			
ATP pre ES	4.99	1.50–16.6	0.009			
Shock pre ES	7.91	3.17–19.7	<0.001	14.1	4.32–46.1	<0.001
Unipolar low voltage (cm^2^)	1.01	0.99–1.02	0.32			
Bipolar low voltage (cm^2^)	1.00	0.99–1.02	0.63			
LP/LAVA	1.37	0.62–3.02	0.44			

Abbreviations: VA: ventricular arrhythmias; HR: hazard ratio; CI: confidence interval; NYHA: New York Heart Association; NSVT: non-sustained ventricular tachycardia; LVEF: ejection fraction; ES: electrical storm; NIDCM: non-ischemic dilated cardiomyopathy; AF: atrial fibrillation; ATP: anti-tachycardia pacing LP: late potential; LAVA: local abnormal ventricular activity.

## Data Availability

The data that support the findings of this study are available on request from the corresponding author, G.B.
